# PGAM5 knockout causes depressive‐like behaviors in mice via ATP deficiency in the prefrontal cortex

**DOI:** 10.1111/cns.14377

**Published:** 2023-08-25

**Authors:** Weiwei Cui, Chunhui Chen, Liya Gong, Junyan Wen, Shanshan Yang, Min Zheng, Baogui Gao, Junxiong You, Xuecong Lin, Yanyu Hao, Zhimin Chen, Ziqi Wu, Liaoming Gao, Jiayu Tang, Zhen Yuan, Xuegang Sun, Linlin Jing, Ge Wen

**Affiliations:** ^1^ Department of Imaging Diagnostics, Nanfang Hospital Southern Medical University Guangzhou China; ^2^ The Second Affiliated Hospital of Guangzhou University of Chinese Medicine Guangzhou China; ^3^ Department of Pharmacy, Nanfang Hospital Southern Medical University Guangzhou China; ^4^ School of Traditional Chinese Medicine Southern Medical University Guangzhou China; ^5^ Centre for Cognitive and Brain Sciences University of Macau Taipa China; ^6^ Traditional Chinese Medicine Integrated Hospital Southern Medical University Guangzhou China

**Keywords:** ATP, dendritic spines, depression, fMRI, PGAM5

## Abstract

**Introduction:**

Major depressive disorder (MDD) affects about 17% population in the world. Although abnormal energy metabolism plays an important role in the pathophysiology of MDD, however, how deficiency of adenosine triphosphate (ATP) products affects emotional circuit and what regulates ATP synthesis are still need to be elaborated.

**Aims:**

Our study aimed to investigate how deficiency of PGAM5‐mediated depressive behavior.

**Results:**

We firstly discovered that PGAM5 knockout (*PGAM5* ^*−/−*^) mice generated depressive‐like behaviors. The phenotype was reinforced by the observation that chronic unexpected mild stress (CUMS)‐induced depressive mice exhibited lowered expression of PGAM5 in prefrontal cortex **(**PFC), hippocampus (HIP), and striatum. Next, we found, with the using of functional magnetic resonance imaging (fMRI), that the functional connectivity between PFC reward system and the PFC volume were reduced in *PGAM5* ^*−/−*^ mice. PGAM5 ablation resulted in the loss of dendritic spines and lowered density of PSD95 in PFC, but not in HIP. Finally, we found that PGAM5 ablation led to lowered ATP concentration in PFC, but not in HIP. Coimmunoprecipitation study showed that PGAM5 directly interacted with the ATP F_1_F_0_ synthase without influencing the interaction between ATP F_1_F_0_ synthase and Bcl‐xl. We then conducted ATP administration to *PGAM5* ^*−/−*^ mice and found that ATP could rescue the behavioral and neuronal phenotypes of *PGAM5* ^*−/−*^ mice.

**Conclusions:**

Our findings provide convincing evidence that PGAM5 ablation generates depressive‐like behaviors via restricting neuronal ATP production so as to impair the number of neuronal spines in PFC.

## Graphical abstract

PGAM5 ablation generates depressive‐like behaviors via restricting ATP production so as to reduce the number of neuronal spines and the expression of psd95 in PFC. And PGAM5 can interact with the ATP F_1_F_0_ synthase without influencing the interaction between PGAM5 and Bcl‐xl.

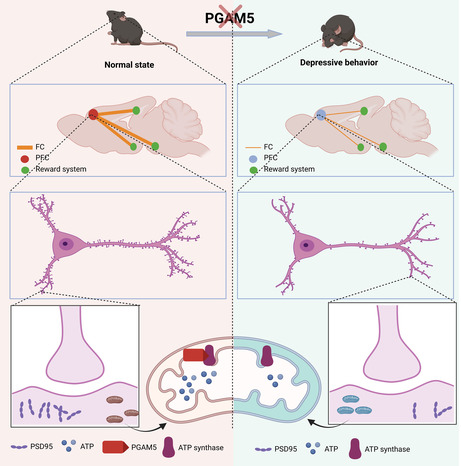

## 1 INTRODUCTION

Major depressive disorder (MDD), the most prevalent psychiatric disease, affects 17% of the world's population. However, its mechanisms remain unclear. Increasing evidence shows that mitochondrial dysfunction plays an important role in the pathogenesis of depression.^1^, ^2^, ^3^ PGAM5, a member of the PGAM family, is a key molecule mediating mitochondrial morphology, mitogenesis, and mitophagy. *PGAM5* ^*−/−*^ mice show Parkinson's symptoms by mediating mitophagy.^4^ However, there are only a few studies on the effects of PGAM5 on the occurrence and development of depression.

Evidence from neuroimaging of patients with major depression and depression animal models have identified the structure and functional activities of some brain regions as the signature of depression. In particular, the prefrontal cortex (PFC), a key brain region for anhedonia, is of vital importance in depression.^5^ Voxel‐based morphometry analysis (VBM) and functional magnetic resonance imaging (fMRI) are the commonly used methods for noninvasive analysis of gray matter volume and brain functional activity in vivo. By VBM, reduced PFC gray matter volume is observed in patients with depression.^6^, ^7^ Functional connectivity is an indicator of the correlation of functional activity between two brain regions. Numerous lines of evidence demonstrate that connectivity between PFC with other regions are also frequently dysregulated in depression.^5^, ^8^ However, the cellular and molecular abnormalities in the PFC of patients with depression and their role in the pathogenesis of depression remain unclear.

Dendritic spines are tiny membrane‐like protrusions from dendritic neurons. In the central nervous system, more than 90% of excitatory synapses are located in dendritic spines,^9^ which are the cell matrix connecting the brain and the main site of information processing in the brain.^10^, ^11^ Dendritic spine pathology is associated with many psychiatric diseases.^11^, ^12^, ^13^, ^14^, ^15^ The formation, growth, and elimination of dendritic spines are commonly dysregulated or disrupted in chronically stressed animals.^16^ The mitochondria in the dendrites of neurons can regulate the dendritic distribution by neuronal activity and the mitochondrial division/fusion.^17^ As a key molecule mediating mitochondrial morphology, we guessesd that PGAM5 can affect the dendritic spines. Therefore, we tested whether dendritic spines were involved in the development of depression in the mouse model in this paper, and Golgi staining was performed to observe dendritic spines in our study.

Mitochondria provide energy in the form of adenosine triphosphate (ATP) to meet cellular needs. A previous study has shown that, after stress modeling, the level of ATP in the mPFC decreases significantly, which can regulate depressive behavior.^18^, ^19^ Zengqiang et al. demonstrated that Calhm2 can regulate depressive behavior by modulating ATP content to affect the number of dendritic spines.^20^ Given that PGAM5 is located on the inner mitochondrial membrane, we hypothesized that PGAM5 may participate in the synthesis of ATP, thereby influencing its level.

## 2 METHODS

### 2.1 Animal

*PGAM5* ^*−/−*^ and C57 mice were purchased from the Shanghai Model Organisms Center, and the Animal Center of Southern Medical University, respectively. They were maintained in the Laboratory Animal Research Center of Nanfang Hospital and housed 4–5 per cage in a specific‐pathogen‐free room at 24 ± 1°C, 40–50% relative humidity, and 12/12 h light/dark cycles and had sufficient food and water. The animal care and experimental procedures were approved by the Institutional Animal Care and Use Committee of Nanfang Hospita.

### 2.2 Behavior test

The protocols for the subsequent tests were slightly modified from those previously described by Gao et al.^21^ To avoid the unfamiliar environment and the investigators causing stress to the mice during the test, they were familiarized with the investigators and the environment for three days before the behavioral tests. The investigators were blinded to the study conditions during the scoring.

#### 2.2.1 Open field test

The open field test (OFT) box is a square box 30 in length, 30 in width, and 35 in depth. At the beginning of the experiment, the box was wiped off with 75% alcohol. After each mouse was placed in the box, its trail was monitored for 5 min using EthoVision software (version 7.0; Noldus).

#### 2.2.2 Forced swim test

A transparent glass container was filled with water approximately 35 cm deep at 28°. In the experiment, the mice were gently placed into the container and allowed to swim for 5 min. The immobility period was recorded, and the immobility state of the mouse is to give up the struggle and just keep the head above the water.

#### 2.2.3 Elevated plus maze test

At the start of the experiment, the field was wiped off with 75% alcohol. The mice were placed in the center of the elevated cross, and their movements were recorded for 5 min. The mouse motion monitoring software used was EthoVision 7.0, Noldus.

### 2.3 CUMS procedure

The chronic unexpected mild stress (CUMS) procedure was modified from that previously described by Yong Zhang et al.^22^ C57 mice were randomly divided into two groups. One group was randomly given the following stimulus: water deprivation, food deprivation, restraint in a 50‐mL tube, and forced swim in cold water (8–10°C). The other group was given no treatment. The specific treatment time schedule and experimental flow is shown in Tables 1 and 2.

**TABLE 1:** The time schedule of chronic unexpected mild stress (CUMS).

The time schedule of CUMS			
Week	day	Stressor	Duration
1	Monday	Food deprivation	24 h
	Tuseday	Water deprivation	24 h
	Wednesday	restraint in a 50‐mL tube	2 h
	Thursday	Forced Swim—cold water swim (8–10°C)	5 min
	Friday	Water and food deprivation	24 h
	Saturday	Restraint in a 50‐mL tube	2 h
	Sunday	Food deprivation	24 h
2	Monday	Water deprivation	24 h
	Tuseday	Forced Swim—cold water swim (8–10°C)	5 min
	Wednesday	Restraint in a 50‐mL tube	2 h
	Thursday	Food deprivation	24 h
	Friday	Water deprivation	24 h
	Saturday	Forced Swim—cold water swim (8–10°C)	5 min
	Sunday	Water and food deprivation	24 h
3	Monday	Restraint in a 50‐mL tube	2 h
	Tuseday	Forced Swim—cold water swim (8–10°C)	5 min
	Wednesday	Water deprivation	24 h
	Thursday	Food deprivation	24 h
	Friday	Restraint in a 50‐mL tube	2 h
	Saturday	Water and food deprivation	24 h
	Sunday	Restraint in a 50‐mL tube	2 h
4	Monday	Water deprivation	24 h
	Tuseday	Food deprivation	24 h
	Wednesday	Restraint in a 50‐mL tube	2 h
	Thursday	Forced Swim—cold water swim (8–10°C)	5 min
	Friday	Water and food deprivation	24 h
	Saturday	Restraint in a 50‐mL tube	2 h
	Sunday	Food deprivation	24 h
5	Monday	Water deprivation	24 h
	Tuseday	Forced Swim—cold water swim (8–10°C)	5 min
	Wednesday	Restraint in a 50‐mL tube	2 h
	Thursday	Food deprivation	24 h
	Friday	Water deprivation	24 h
	Saturday	Forced Swim—cold water swim (8–10°C)	5 min
	Sunday	Water and food deprivation	24 h

**TABLE 2:** The experimental flow of chronic unexpected mild stress (CUMS).

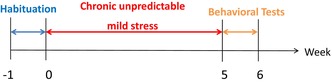

### 2.4 Golgi staining

The FD Rapid Golgi Stain Kit was used for Golgi staining where the general process was as follows. First, after anesthesia, the mouse brain tissue was removed and soaked in an equal mixture of reagents A and B for approximately 2 weeks. They were then transferred to solution C and soaked for 3 days. Coronal sections (100 μm) were cut after embedding in the tissue freezing medium (TFM). The specific staining steps were performed following the manufacturer's instructions.

### 2.5 Nissl's staining

After anesthesia, mice were perfused with PBS and 4% paraformaldehyde. Brain tissue was removed and soaked overnight in 4% paraformaldehyde. The tissue was soaked in 15% sucrose solution overnight and then transferred to 30% sucrose solution. Coronal sections (15 μm) were cut after embedding in OCT. Cells were fixed with 4% paraformaldehyde for more than 10 min. The samples were then stained with Nissl staining solution (Beyotime, C0117) for 10 min. After dehydration, images of stained brain slices were acquired using a scanner (kfbio).

### 2.6 Western blotting

The anesthetized mice were perfused with PBS, and brain tissues removed from the frontal lobe and hippocampus (HIP) were isolated. WB lysate (Beyotime, P0013) or NP40 lysate (Beyotime, P0013F) was then added to the tissue at a ratio of 100 μL/mg. After vortexing (60 Hz, 60 s) and centrifugation (12,000 × *g*, 4°C, 15 min), a bicinchoninic acid (BCA) protein assay kit (Beyotime Biotechnology, P0009) was used to quantify the protein lysate in the 96‐well plates (SORFA Life Science). Thirty micrograms of each sample were transferred to a polyvinylidene fluoride (PVDF) membrane after separation by perdodecyl sulfate polyacrylamide gel electrophoresis and then blocked with 5% BSA at 25°C for 2 h. The primary antibody was incubated at 4°C overnight, and immunolabeling was performed using rabbit anti‐Pgam5 (1:1000 dilution, Abcam, ab108319), rabbit anti‐Bcl‐xL (1:1000 dilution, Cell Signaling, 54H6), mouse anti‐GAPDH (1:1000 dilution, Proteintech, 60,004‐1‐lg), rabbit anti‐ATP‐β subunit (1:5000 dilution, Abcam, ab170947), rabbit anti‐Psd95 (1:1000 dilution, Proteintech, 20,665‐1‐AP‐50UL), and mouse anti‐Snap25 (1:1000 dilution, Proteintech, 60,159‐1‐IG‐50UL) antibodies. The results were visualized by chemiluminescence (GE Healthcare Bio‐Science). Quantitative analysis of the images was performed using Imaging J software.

### 2.7 Immunoprecipitation

The lysate was added to protein A/G magnetic beads (Bimake, B23201) for 1 h, and then the magnetic beads were discarded. The supernatant was incubated with 2.5 μg rabbit anti‐Pgam5 (Abcam, ab126534) and 2 μg rabbit anti‐Bcl‐xL (Cell Signaling, 54H6) antibodies overnight at 4°C with 360°C agitation. The antigen antibody solution was then incubated with magnetic beads for 4 h. After reaction with the 2× loading buffer at 98°C for 8 min, 20 μL of the sample was added to each well, and western blotting analysis steps were repeated as described above.

### 2.8 ATP detection

An enhanced ATP Assay Kit (Beyotime, S0027) was used to detect the ATP levels. The general process was as follows: 100 μL ATP detection working solution was added to each sample or standard well for a 3–5 min reaction. Then, 20 μL of sample or standard was added to the test hole, quickly mixed with a gun (micropipette), and relative light unit (RLU) was determined using a luminometer.

### 2.9 qPCR

Total RNA was extracted, isolated, and purified from the tissues using the Tissue RNA Purification Kit PLUS (EZB‐RN001‐PLUS). Briefly, we used HiScript III RT SuperMix for qPCR kit (R323‐01, China) for cDNA synthesis and quantitative RT‐PCR using 2× SYBR Green qPCR Master Mix Kit (B21203‐25 mL). The sequences of the qPCR primers used to quantify mRNA expression in the present study were mouse Pgam5‐5F: 5′‐CCTGACCAAGTGCCTCAAACA‐3′, mouse Pgam5‐5F: 5′‐ CCTGTTCCCGACCTAATGGT‐3′, mouse GAPDH‐5F: 5′‐AGGTCGGTGTGAACGGATTTG‐3′, and mouse GAPDH‐3R‐5′‐TGTAGACCATGTAGTTGAGGTCA‐3′.

### 2.10 Magnetic resonance scanning

Mice were anesthetized with 3% isoflurane before scanning. All MRI scans were performed using a 7T Bruker scanner (Pharmascan 70/16, US). During the scan, mice were fixed on a plastic cradle and monitored for breathing and circulation. For structural MRI, the Turbo RARE sequence was used to scan 3D T2‐weighted images (3D‐T2WI). For resting‐state fMRI, we used a spin‐echo echo‐planar imaging (SE‐EPI) sequence with 500 time points, TR/TE: 1500/21.2 ms, matrix: 96 × 96, flip angle: 90°, voxel size: 0.7 × 0.156 × 0.156 mm, 15 slices, and slice thickness: 0.7 mm.

### 2.11 MRI preprocessing and analysis

MRI process and analysis were performed as previously described.^23^ For VBM analysis, all mice 3D T2‐WI images were preprocessed using SPM12 software (Wellcome Department of Cognitive Neurobiology, University College of London, UK), which included linear registration, segmentation, normalization, Jacobian modulation, and smoothing.

For rs‐fMRI analysis, we first discarded 10 volumes of fMRIs. After correcting the slice timing and head motif for the rest of the volumes, they were spatially normalized to the template established by 3D T2‐WI images. Then, to smooth the images, an 8‐mm full width at half maximum (FWHM) Gaussian kernel was used. Signal regression and motion vectors were used to correct for systemic noise. Next, the seed region of interest (ROI) was obtained from the VBM results. Second, the resting‐state functional connectivity map between the PFC and the reward system was calculated using the Pearson correlation coefficient. Fisher Z‐transformation was performed to increase the normality of the functional connectivity data. After statistical analysis of functional connectivity values using GraphPad Prism 8 (La Jolla, CA, USA), *t*‐values reflecting the differences in functional connectivity were converted into a matrix using MATLAB software (The MathWorks Inc).

### 2.12 Statistical analysis

Normality and homoscedasticity were evaluated using the Shapiro–Wilk or Kolmogorov–Smirnov and Levene tests, respectively. For comparisons between datasets with normal distribution, unpaired Student's *t*‐tests or one‐way analysis of variance (ANOVA) were performed with Dunnett's test for post hoc comparisons, with KO as the control group, using GraphPad Prism 8 and IBM SPSS Statistics Version 26.0. Datasets without a normal distribution were analyzed for significance using the Mann–Whitney *U*‐test. Differences were considered statistically significant at *p* < 0.05. Statistical analyses results are reported in Table S1.

## 3 RESULTS

### 3.1 The expression of PGAM5 reduced in CUMS mice

**FIGURE 1:**
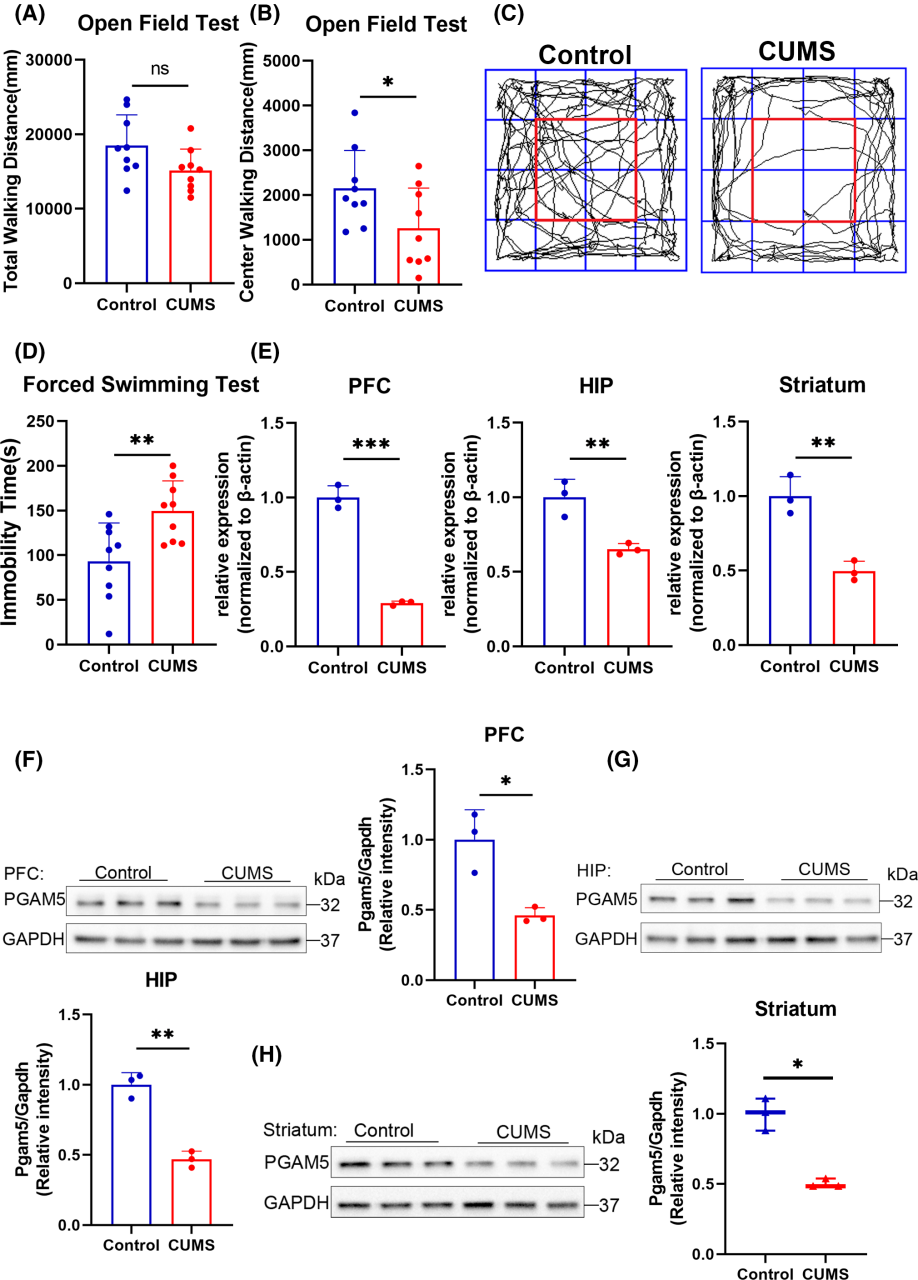
The expression of PGAM5 reduced in chronic unexpected mild stress **(**CUMS) mice. (A–C) Total walking distance and center walking distance in the open field test (OFT). After CUMS, the mice exhibited reduced central walking distance (B, C) without affecting the total locomotive activity (A, C). (D) Forced swim test (FST) immobile time. *CUMS* mice showed increased immobility time in FST compared to the Control (Control *n* = 9, CUMS *n* = 9). (E) The mRNA level of pgam5 in prefrontal cortex (PFC), hippocampus (HIP), and striatum decreased significantly in CUMS mice (Control *n* = 3, CUMS *n* = 3). (F, G, H) Western blotting and analysis of pgam5 expression in PFC, HIP, and striatum. The expression of pgam5 was makably reduced in CUMS mice. Bar plots represent mean ± SD, while boxplots represent the minimum, median, and maximum. ***p < 0.001, **p < 0.01, *p < 0.05 and ns, not significant.

PGAM5, a mitochondrial membrane protein, is of vital importance for the mitochondrial homeostasis. To investigate the relationship between PGAM5 and depression, we first used CUMS‐treated mice, which displayed reduced central walking distance in the OFT (*p* < 0.05 for Control vs. CUMS; Figure 1A–C) and increased immobility time in the forced swim test (FST) (*p* < 0.01 for Control vs. CUMS; Figure 1D). Compared with the control, CUMS‐treated mice showed decreased expression of PGAM5 protein (*p* < 0.05 for Control vs. CUMS in PFC, Figure 1F; *p* < 0.01 for Control vs. CUMS in HIP, Figure 1H; *p* < 0.05 for Control vs. CUMS in Striatum, Figure 1G) and mRNA (*p* < 0.001 for Control vs. CUMS in PFC, *p* < 0.01 for Control vs. CUMS in HIP, *p* < 0.01 for Control vs. CUMS in Striatum, Figure 1E) in the PFC, HIP, and striatum.

### 3.2 Depressive‐like behaviors in *PGAM5* ^*−/−*^ mice

**FIGURE 2:**
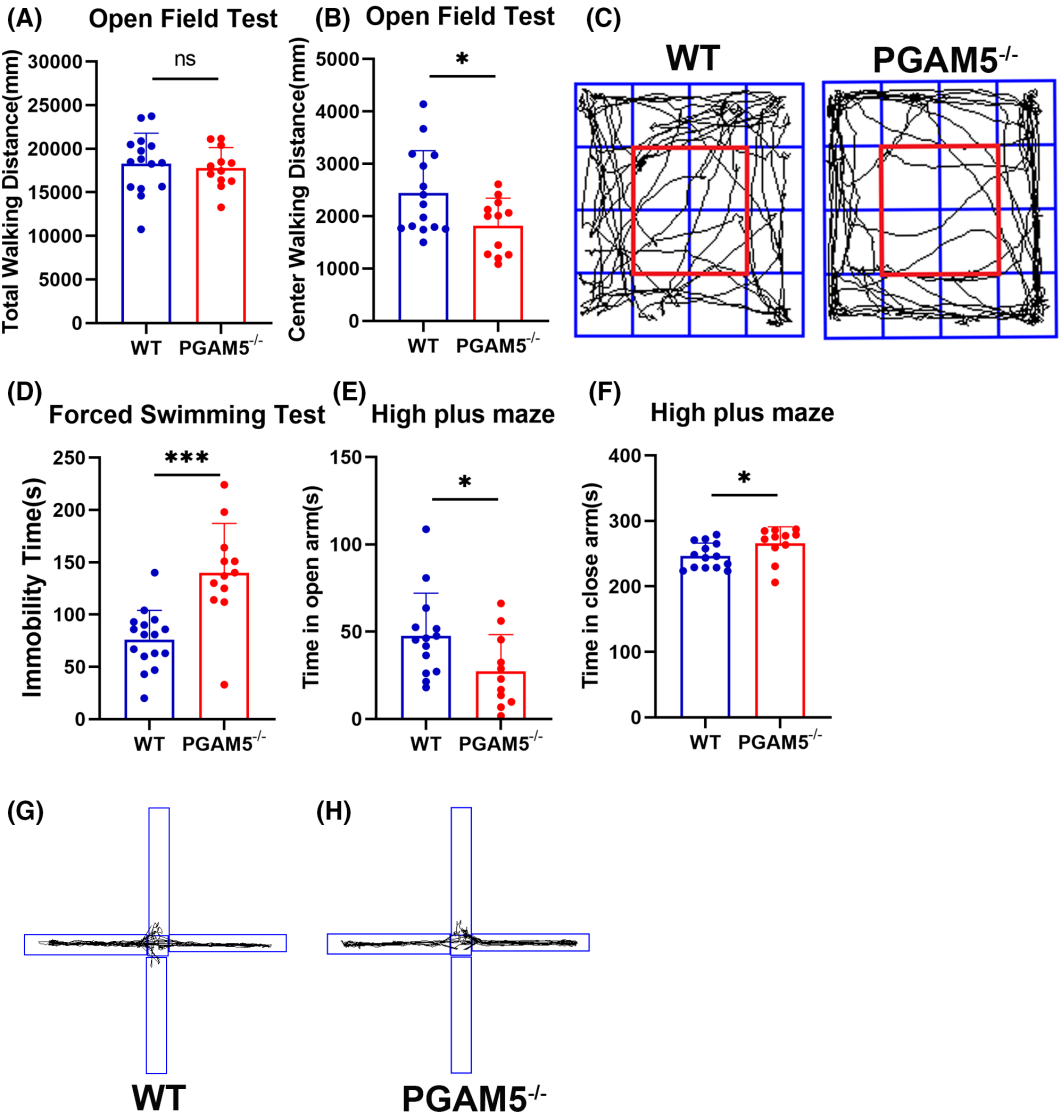
*PGAM5* ^*−/−*^ mice display a depression‐like phenotype. (A–C) Total walking distance and center walking distance in the open field test (OFT). After PGAM5 knockout, the mice exhibited reduced central walking distance (B, C) without affecting the total locomotive activity (A, C) (wildtype (WT) *n* = 15, *PGAM5* ^*−/−*^ *n* = 12). (D) Forced swim test (FST) immobile time. *PGAM5* ^*−/−*^ mice showed increased immobility time in FST compared to the WT (WT *n* = 16, *PGAM5* ^*−/−*^ *n* = 12). (E–H) Time in close arm and open arm in elevated plus maze (EPM). *PGAM5* ^*−/−*^ mice spent more time in the closed arms (E) and less time in the open arms (F). The vertical and horizontalaxis of the graph are the open arm and close arm, respectively (WT *n* = 14, *PGAM5* ^*−/−*^ *n* = 11). Data are expressed as the mean ± SD. ***p < 0.001, **p < 0.01, *p < 0.05 and ns, not significant.

To futher investigate the relationship, we used *PGAM5* ^*−/−*^ mice to test the depressive behavior of *PGAM5* ^*−/−*^ mice and wildtype (WT) mice. We observed that, after PGAM5 knockout, the mice exhibited reduced central walking distance (*p* < 0.05 for WT vs. *PGAM5* ^*−/−*^; Figure 2B,C) in the OFT without affecting the total locomotive activity (*p* > 0.05 for WT vs. *PGAM5* ^*−/−*^; Figure 2A,C) and increased immobility time in the FST (*p* < 0.001 for WT vs. *PGAM5* ^*−/−*^; Figure 2D). In addition, *PGAM5* ^*−/−*^ mice spent more time in the closed arms and less time in the open arms in the elevated plus maze (EPM), anxiety‐related behavior tests (*p* < 0.05 for WT vs. *PGAM5* ^*−/−*^ in the closed arms, Figure 2F–H; *p* < 0.05 for WT vs. *PGAM5* ^*−/−*^ in the open arms, Figure 2E,G,H). These data suggest that PGAM5 is closely related to depressive behavior.

### 3.3 Abnormal MRI pattern of *PGAM5* ^*−/−*^ mice

**FIGURE 3:**
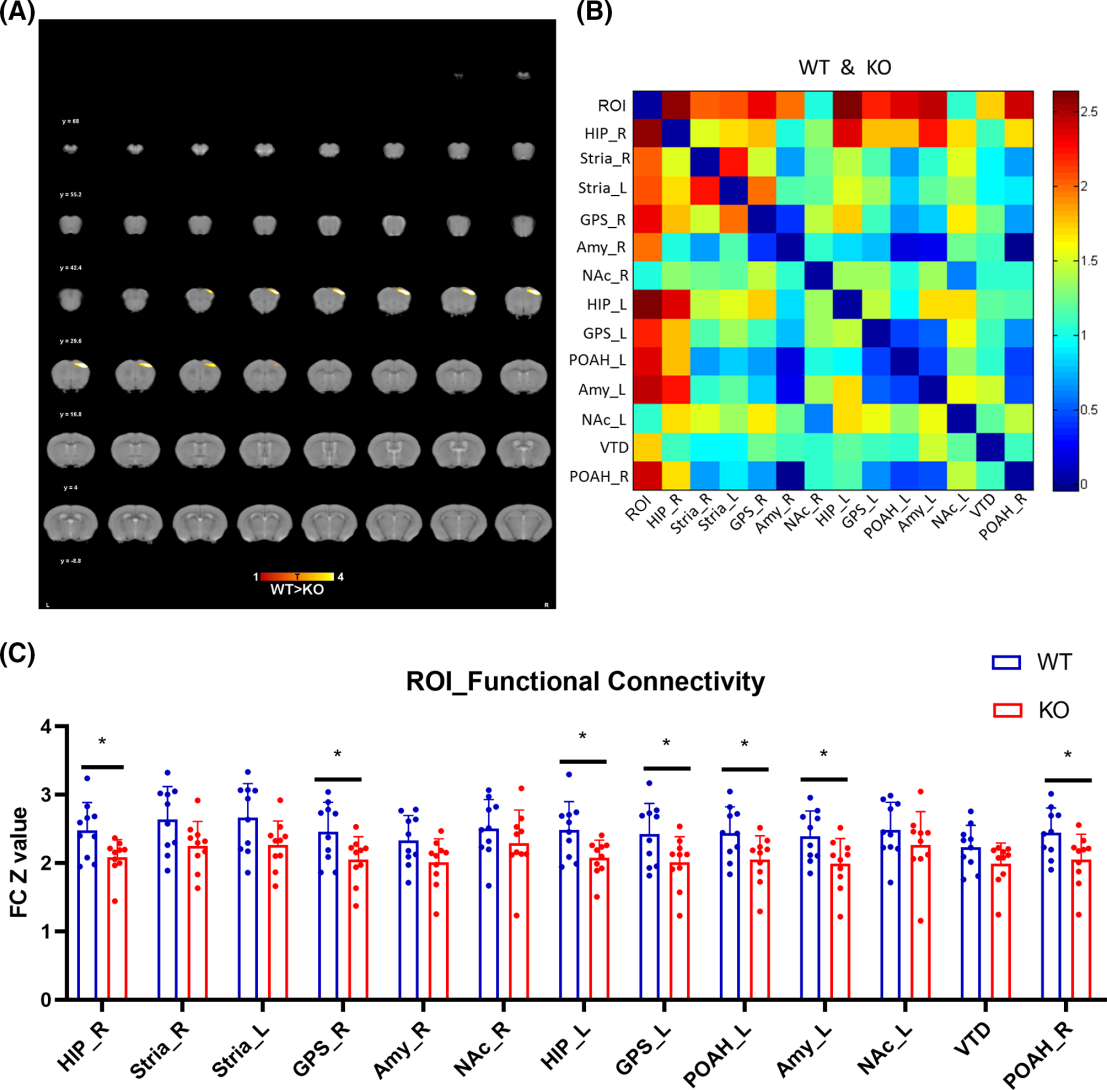
Abnormal MRI pattern of *PGAM5* ^*−/−*^ mice. (A) The highlight regions were decreased volume of gray matter in the *PGAM5* ^*−/−*^ mice (KO) compared with the WT shown on the coronal plane. (*p* < 0.001, with FDRc‐corrected; WT *n* = 25, *PGAM5* ^*−/−*^ *n* = 31). According to the atlas, this region belongs to PFC. And no increased region was detected in the KO mice compared to the WT. (B, C) The PFC was selected as a seed region of interest (ROI) for further functional connectivity analysis. The T matrix is a matrix of *t*‐statistic values for WT and KO group differences in functional connectivity between paird regions of ROI reward system. Each element in the matrix indexes the paired regions. With the color of the element closer to red, the group difference in functional connectivity is greater. The T value matrix (B) of state brain connectivity correlation among the ROI and right HIP (HIP_R), right striatum (Stria_R), left striatum (Stria_L), right globus pallidus (GPS_R), right amygdala (Amy_R), right nucleus accumbens (NAc_R), left HIP (HIP_L), left globus pallidus (GPS_L), left hypothalamus (POAH_L), left amygdala (Amy_L), left nucleus accumbens (NAc_L), ventral tegmental decussation (VTD), and right hypothalamus (POAH_R) between WT and KO. (C) The bar graph with functional connectivity value between other regions with the PFC in both groups *(p* < 0.05, with FDRc‐corrected; WT *n* = 10, *PGAM5* ^*−/−*^ *n* = 9). Data are expressed as mean ± SD. *p < 0.05.

To explore the role of PGAM5 in regulating depressive behavior, we conducted MRI to analyze the brain volume between WT and *PGAM5* ^*−/−*^ mice. Interestingly, we found that the PFC volume in *PGAM5* ^*−/−*^ mice was markedly decreased (*p* < 0.001 for WT vs. KO, with cluster‐level false discovery rate (FDRc)‐corrected; Figure 3A). Therefore, the PFC was selected as a seed ROI for further functional connectivity analysis, and this region has emerged as one of the most consistently impaired regions in MDD.^5^ Because the HIP, striatum (Stria), globus pallidus (GPS), amygdala (Amy), nucleus accumbens (NAc), hypothalamus (POAH), and ventral tegmental decussation (VTD), as part of the reward system, play vital roles in the regulation of emotion and depression, we chose these regions to analyze the functional connectivity with the PFC. Compared toWT, decreased PFC functional connectivity was found in the bilateral HIP, bilateral GPS, left Amy, and bilateral POAH in *PGAM5* ^*−/−*^ mice (*p* < 0.05 for WT vs. KO in bilateral HIP, bilateral GPS, left Amy, and bilateral POAH; Figure 3B,C).

### 3.4 *PGAM5* ^*−/−*^ mice display damaged neuron structure

**FIGURE 4:**
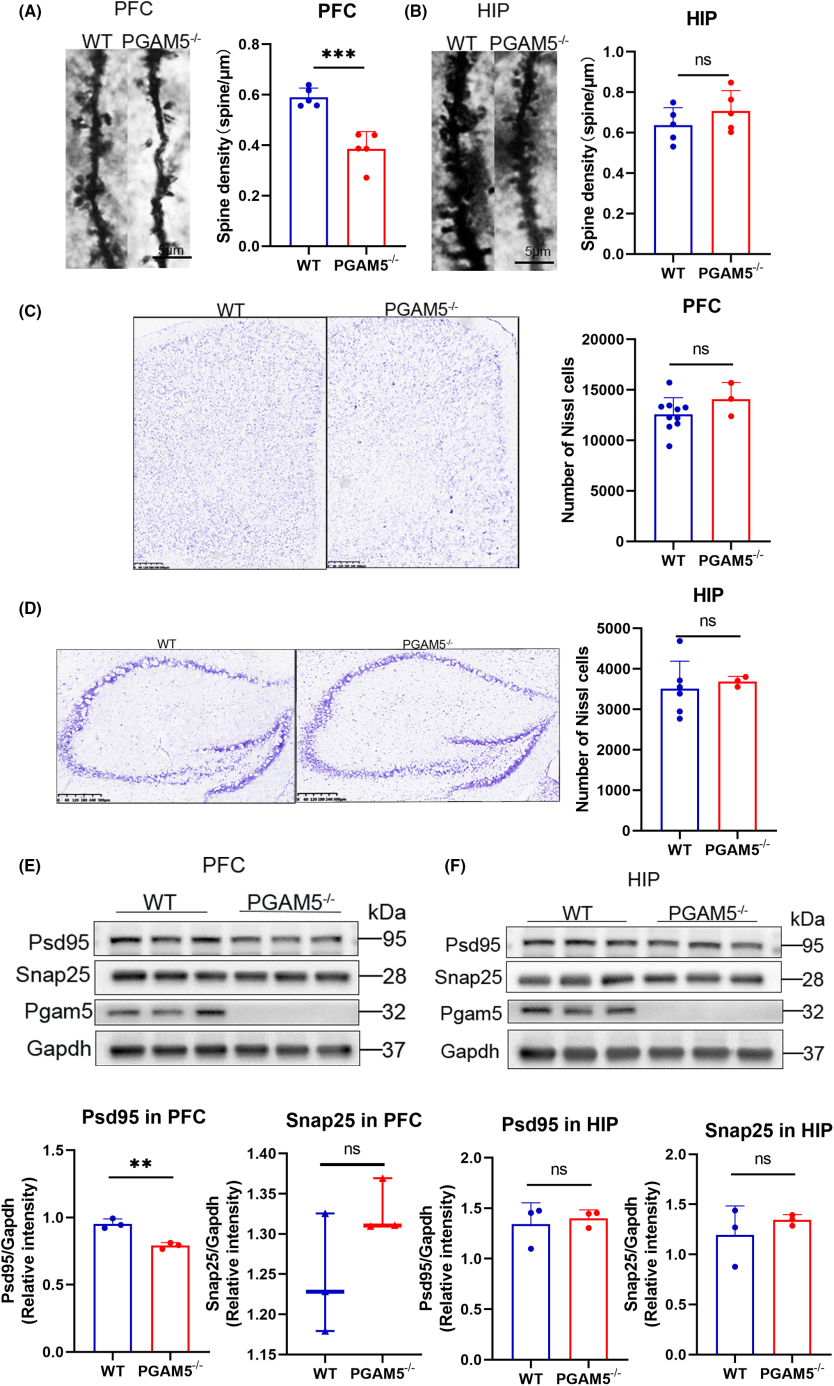
*PGAM5* ^*−/−*^ mice displayed damaged neuron structure. (A, B) Confocal micrographs of dendrites from WT and KO mice and bar graphs with quantification of spine density. KO mice had significantly fewer dendritic spines than the WT mice in the PFC (A), but there were no group differences in the hippocampus (B) (WT *n* = 5; *PGAM5* ^*−/−*^ *n* = 5). (C, D) The number of positive cells in Nissl staining. No group differences in KO and WT mice were detected in either the PFC (C) or the hippocampus (D) (PFC, WT *n* = 10 and *PGAM5* ^*−/−*^ *n* = 3; hippocampus, WT *n* = 6 and *PGAM5* ^*−/−*^ *n* = 3). (E, F) Western blotting and analysis of psd95 and snap25 expression. The level of psd95, a marker of postsynaptic density, was decreased in KO mice in the PFC, but there were no group differences in the hippocampus (E). Immunoblots of snap25, a synaptic protein showed no significant difference between WT and KO mice in either the PFC or hippocampus (F) (WT *n* = 3; *PGAM5* ^*−/−*^ *n* = 3). Bar plots represent mean ± SD, while boxplots represent the minimum, median, and maximum. ***p < 0.001, **p < 0.01, *p < 0.05 and ns, not significant.

The Nissl body of CUMS‐treated mice is reduced in the HIP and PFC compared with the control.^24^, ^25^, ^26^, ^27^, ^28^, ^29^ Furthermore, some studies have confirmed that, after CUMS, mice tend to display fewer dendritic spines in the HIP and in the PFC.^26^, ^30^, ^31^, ^32^, ^33^, ^34^ To examine how PGAM5 is related to the structure of neurons, we conducted Nissl and Golgi staining of *PGAM5* ^*−/−*^ and WT mice. We found that the *PGAM5* ^*−/−*^ mice had fewer dendritic spines than the WT mice in the PFC (*p* < 0.001 for WT vs. *PGAM5* ^*−/−*^; Figure 4A), but there were no group differences in the HIP (*p* > 0.05 for WT vs. *PGAM5* ^*−/−*^; Figure 4B). In addition, no group differences in Nissel bodies in *PGAM5* ^*−/−*^ and WT mice were observed in either the PFC or the HIP (*p* > 0.05 for WT vs. *PGAM5* ^*−/−*^; Figure 4C,D). The level of psd95, a marker of postsynaptic density, was decreased in *PGAM5* ^*−/−*^ mice in the PFC (*p* < 0.01 for WT vs. *PGAM5* ^*−/−*^; Figure 4E), but there were no group differences in the HIP (*p* > 0.05 for WT vs. *PGAM5* ^*−/−*^; Figure 4F). To understand the impact of PGAM5 on synaptic activities, we assessed the level of synaptic protein snap25, and immunoblots of snap25 showed no significant difference between WT and *PGAM5* ^*−/−*^ mice in either the PFC or HIP (*p* > 0.05 for WT vs. *PGAM5* ^*−/−*^; Figure 4E,F).

### 3.5 PGAM5 ablation leads to reduced ATP concentration by interacting with F1F0 ATP synthase

**FIGURE 5:**
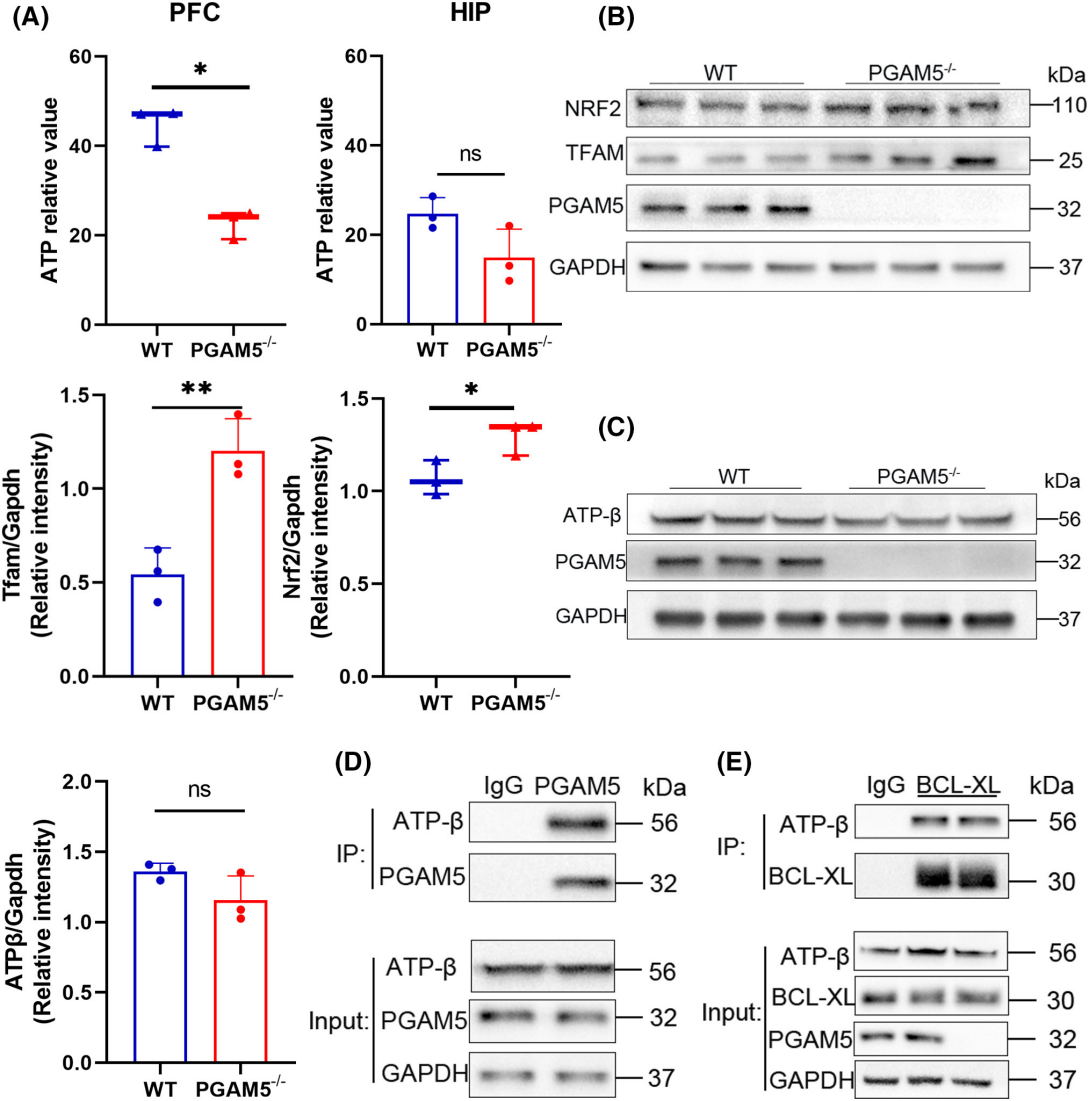
PGAM5 ablation led to reduced ATP concentration by interacting the F_1_F_0_ ATP synthase. (A) The ATP relative value of PFC was higher in WT mice than KO mice (WT *n* = 3; *PGAM5* ^*−/−*^ *n* = 3). (B) KO mice had higher expression of Nrf2 and Tfam in PFC. (C) The expression of the F_1_F_0_ ATP synthase β subunit (ATP‐β) did not alter significantly in PFC. (D) The coimmunoprecipitation of PGAM5 and ATP‐β. PGAM5 pull down the ATP‐β. (E) The effect of PGAM5 on the interaction between Bcl‐xl and ATP‐β. Bcl‐xl pull down the ATP‐β. Bar plots represent mean ± SD, while boxplots represent the minimum, median, and maximum. ***p < 0.001, **p < 0.01, *p < 0.05 and ns, not significant.

Given that ATP can have a rapid antidepressive effect and has a close relationship with dendritic spines, we hypothesized that PGAM5 may regulate ATP levels to influence depressive behavior. First, ATP levels were measured, and we found that the PFC had higher ATP concentrations in WT mice than *PGAM5* ^*−/−*^ mice (*p* < 0.05 for WT vs. *PGAM5* ^*−/−*^ in PFC; *p* > 0.05 for WT vs. *PGAM5* ^*−/−*^ in HIP; Figure 5A). PGAM5 plays an important role in mitochondrial biogenesis.^4^ Therefore, we examined the expression of nrf2 and TFAM in the PFC and found that knockdown of PGAM5 improved mitochondrial biogenesis (*p* < 0.01 for WT vs. *PGAM5* ^*−/−*^, NRF2; *p* < 0.05 for WT vs. *PGAM5* ^*−/−*^, TFAM; Figure 5B). The ATP level was still lower in *PGAM5* ^*−/−*^ mice, even though mitochondrial biogenesis was higher. Therefore, we hypothesized that PGAM5 regulates key enzymes involved in ATP synthesis. Because PGAM5 and F_1_F_0_ ATP synthase are both mitochondrial inner membrane proteins, we performed co‐immunoprecipitation assays and observed that PGAM5 interacted with the F_1_F_0_ ATP synthase β subunit (Figure 5D), and the level of the F_1_F_0_ ATP synthase β subunit was lower in *PGAM5* ^*−/−*^ mice (*p* > 0.05 for WT vs. *PGAM5* ^*−/−*^; Figure 5C). Jonas et al. reported that Bcl‐xL interacts directly with the β subunit of F_1_F_0_ ATP synthase, which regulates synaptic efficacy.^35^ Furthermore, in 2020, Chen et al. reported that PGAM5 dephosphorylates BCL‐xL to inhibit apoptosis.^36^ To investigate whether the observed interaction was general through BCL‐xL, we performed co‐immunoprecipitation assays after knocking out PGAM5 and found no difference in the combination of BCL‐xL and F_1_F_0_ ATP synthase β subunit (Figure 5E). Thus, we conclude that PGAM5 regulates ATP levels by interacting with F_1_F_0_ ATP synthase without influencing the interaction between ATP F_1_F_0_ synthase and Bcl‐xl.

### 3.6 ATP injection rescues the behavioral and neuronal phenotypes of *PGAM5* ^*−/−*^ mice

**FIGURE 6:**
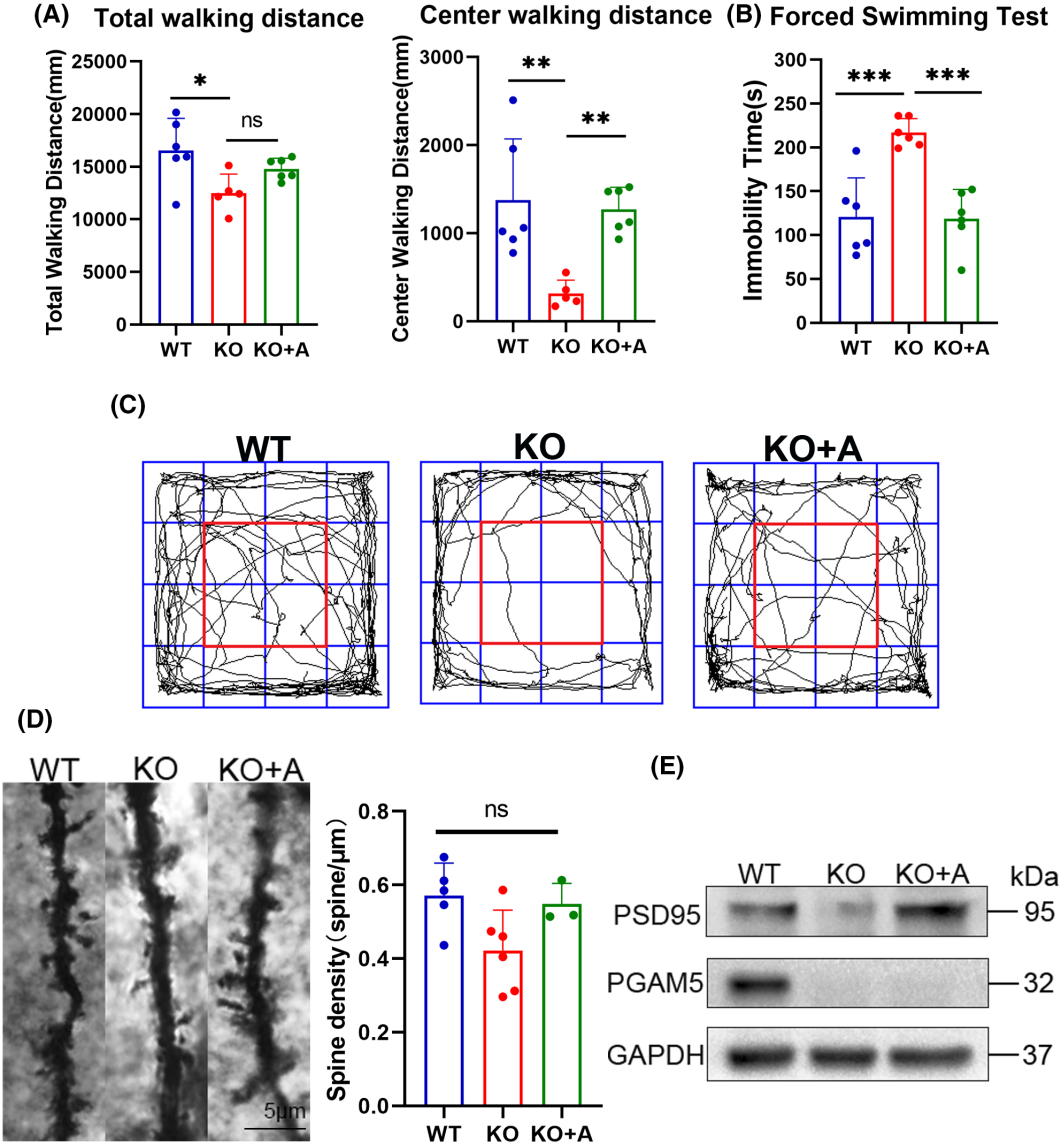
ATP injection rescued behavioral and neuronal phenotypes. (A) Open field test total and center walking distance. (B) Forced swim test immobile time (WT, *n* = 6; KO, *n* = 6; KO + A, *n* = 6). (C) Diagram in the open field test, red frame is the center area. ATP supplementation significantly increased the central duration in the OFT and exhibited a struggling time extension in the FST. (D) Confocal micrographs of dendrites of PFC from WT and KO mice and bar graphs with quantification of spine density (WT, *n* = 5; KO, *n* = 6; KO + A, *n* = 3). (E) The expression of psd95 in PFC. Dendritic spine loss and the reduction of psd95 induced by PGAM5 deficiency were markedly reversed by ATP injection. Normality and homoscedasticity were evaluated using Kolmogorov–Smirnov tests followed by Dunnett's post hoc test. Data are expressed as mean ± SD. ***p < 0.001, **p < 0.01, *p < 0.05 and ns, not significant. KO, *PGAM5* ^*−/−*^; KO + A, *PGAM5* ^*−/−*^ + ATP; WT, wildtype.

As ATP has antidepressant effects and PGAM5 could affect the level of ATP, we studied whether ATP administration could normalize the depressive behavior and neuronal structure injury in *PGAM5* ^*−/−*^ mice. To achieve this, *PGAM5* ^*−/−*^ mice were treated with ATP via intraperitoneal injections of 125 mg/kg/day for 7 days. Following treatment, *PGAM5* ^*−/−*^ mice exhibited a struggling time extension in the FST (*p* < 0.001 for KO vs. KO + A; Figure 6C). In addition, ATP supplementation significantly increased the central duration (*p* < 0.01 for KO vs. KO + A; Figure 6B) in the OFT. To determine how ATP normalized the depression‐like phenotype of *PGAM5* ^*−/−*^ mice, we examined the morphology and function of the neuronal spines. We observed that dendritic spine loss and the reduction of psd95 induced by PGAM5 deficiency were reversed by ATP injection (*p* > 0.05 for experimental group; Figure 6D,E).

## 4 DISCUSSION

In summary, we observed that not only was PGAM5 expression reduced in CUMS mice but adult PGAM5‐deficient mice displayed depressive‐like behavior. Using fMRI, we found that the PFC volume of *PGAM5* ^*−/−*^ mice and PFC reward system connectivity decreased, further confirming the role of PGAM5 in depression. To examine whether PGAM5 mediates the depressive behavior and MRI findings through the alteration of neuronal structure, we conducted Golgi and Nissl stainings and concluded that the loss of dendritic spines may be the key. We used a *PGAM5* ^*−/−*^ mouse model and validated that the number of spines significantly decreased by reducing ATP levels and could be rescued by ATP administration. Co‐immunoprecipitation assays were also performed, and we discovered that PGAM5 can interact with ATP F_1_F_0_ synthase directly.

PGAM5 is closely related to mitophagy, mitochondrial fission, mitochondrial biogenesis, and programmed cell death,^4^ and PGAM5 may regulate depressive behavior. However, this possibility may be small for several reasons. First, recent studies have shown that the level of mitophagy is higher in patients with MDD than in the control.^37^ Since PGAM5 plays a vital role in mitophagy, the deletion of PGAM5 should lead to an antidepressive phenotype. We further corroborated that the levels of nrf2 and TFAM in the PFC, a marker of mitochondrial biogenesis, are increased as opposed to the study that states nrf2 in MDD patients is reduced compared to the control.^38^ Third, preclinical studies have demonstrated that stress or depression lead to atrophy and cell loss in limbic brain structures that are critical.^39^ However, PGAM5, the key molecule in the conversion from mitochondrial dynamics to programmed cell death, has an antidepressive effect, arguing against this scenario. In summary, PGAM5 might regulate depressive behavior by other means, and there is a great possibility that PGAM5 regulates depressive behavior by directly influencing the level of ATP. Our results suggest that this is the case.

Dendritic spines, the key structural communication hub of neurons from different brain regions, typically receive input from axons at the postsynaptic membrane. Functional connectivity is a crucial tool for measuring the activity relationships between different brain regions. In our study, reduced connectivity between the PFC and its reward system was observed in *PGAM5* ^*−/−*^ mice, which may result from the loss of dendritic spines. The decrease in PFC volume in *PGAM5* ^*−/−*^ mice observed by VBM analysis may result from neural synapse's loss. It is well established that depressive disorders are associated with structural changes and neuronal atrophy. There is also a synaptogenesis hypothesis of depression stating that dysregulated synaptogenesis contributes to aberrant neural circuit activity that underlies depressive behavior.^40^ Thus, our results strongly support these findings.

In neurons, mitochondria are located in dendritic shafts and spines, which play a pivotal role in synaptic plasticity. Li reported that a reduction in dendritic mitochondrial content gives rise to loss of dendritic spines, whereas the number of dendritic spines increases in the wake of mitochondrial accumulation.^17^ As the most direct source of energy produced by the mitochondria in living organisms, ATP may mediate the mitochondrial effect on dendritic spines. In astrocytes, ATP can be released out the cell through Calhm2, which regulates hippocampal spine number.^20^ Here, we used a *PGAM5* ^*−/−*^ mouse model and validated that the number of spines significantly decreased by reducing ATP levels and could be rescued by ATP administration.

ATP production by oxidative phosphorylation is a key method by which mitochondria provide energy to the cells. ATP synthase is a key enzyme involved in oxidative phosphorylation, and its interaction with PGAM5 may be an important reason for the decrease in ATP levels following PGAM5 knockout. However, the mechanism through which the interaction between PGAM5 and ATP synthase affects ATP synthesis requires further investigation. In the brain, ATP is not only a cellular energy fuel but also essential for normal neuronal processes, including the maintenance of ion gradients on neuronal membranes, accumulation of neurotransmitters in vesicles, release of neurotransmitters, and movement of receptors and ion channels into and out of the cell surface.^41^ This may be another reason for the decreased prefrontal volume and functional connectivity with the reward system in KO mice.

Our results suggest that ATP treatment or enhanced PGAM5 function are possible therapeutic avenues for the treatment of MDD. However, because of the difficulty in obtaining genetic mice and the rarity of animal brain tissue, the number of experimental samples in this study was small, so this view needs to be verified by further experiments.

## CONFLICT OF INTEREST STATEMENT

The authors declare that the research was conducted in the absence of any commercial or financial relationships that could be construed as potential conflicts of interest.

## Supporting information


Data S1.

Table S1.

## Data Availability

The original data presented in this study have been included in the manuscript and tables. Further inquiries can be directed to the corresponding author.
