# Supplementary material for: PGAM5 knockout causes depressive‐like behaviors in mice via ATP deficiency in the prefrontal cortex
Source: CNS Neurosci Ther. 2023 Aug 25;30(2):e14377. doi: 10.1111/cns.14377 (PMC10848067; doi:10.1111/cns.14377)

Full unedited blot for Figure1F-PFC

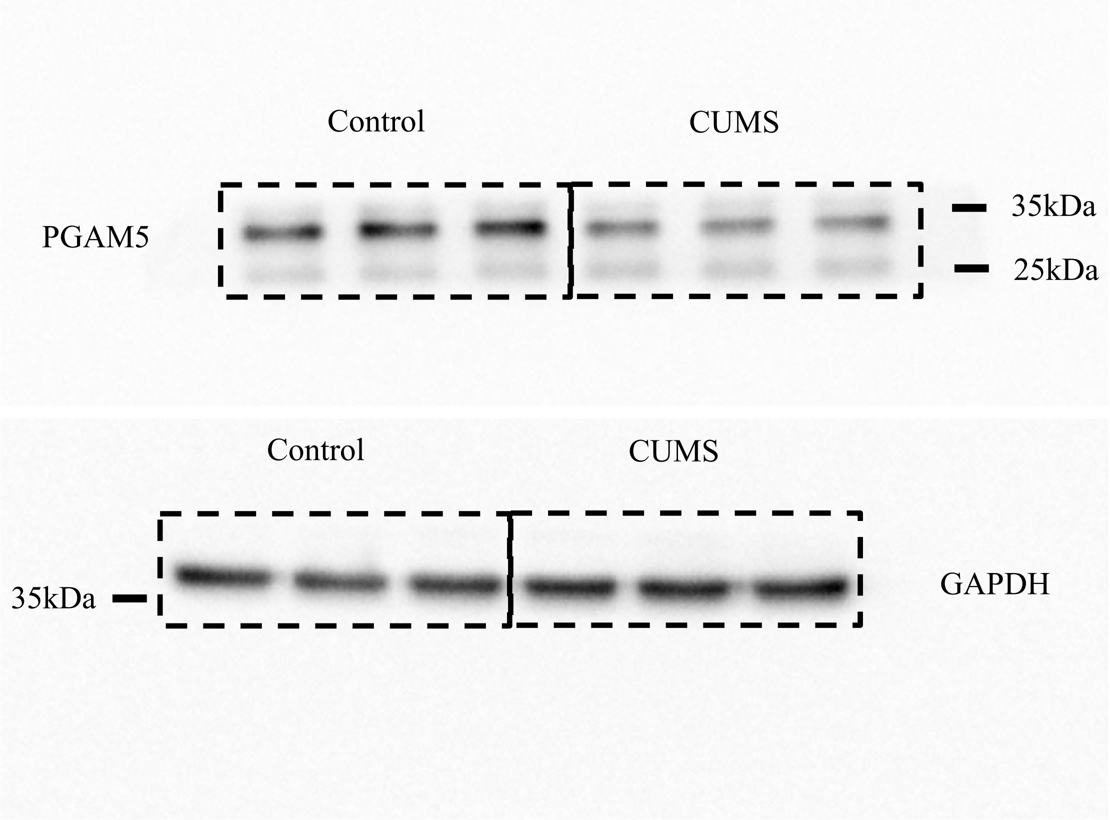

Full unedited blot for Figure1F-HIP

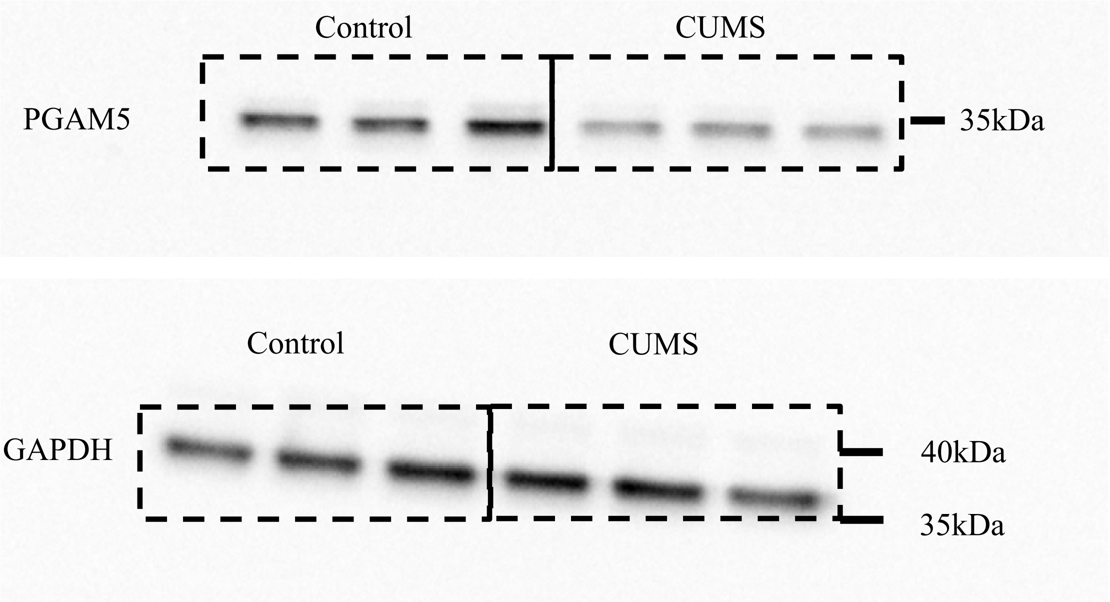

Full unedited blot for Figure1F-Striatum

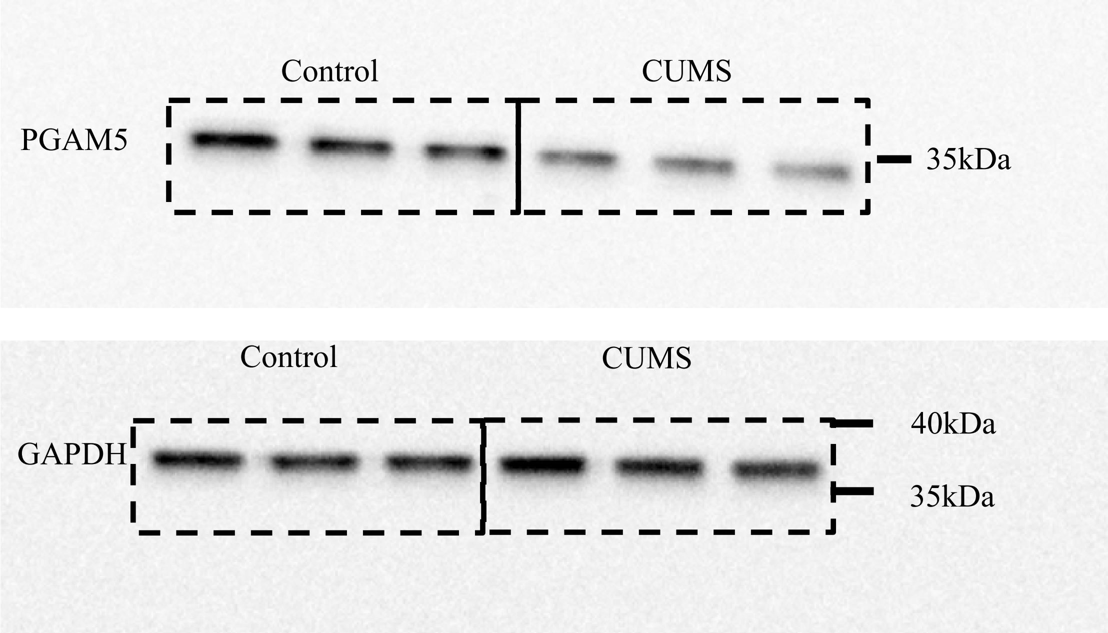

Full unedited blot for Figure4E

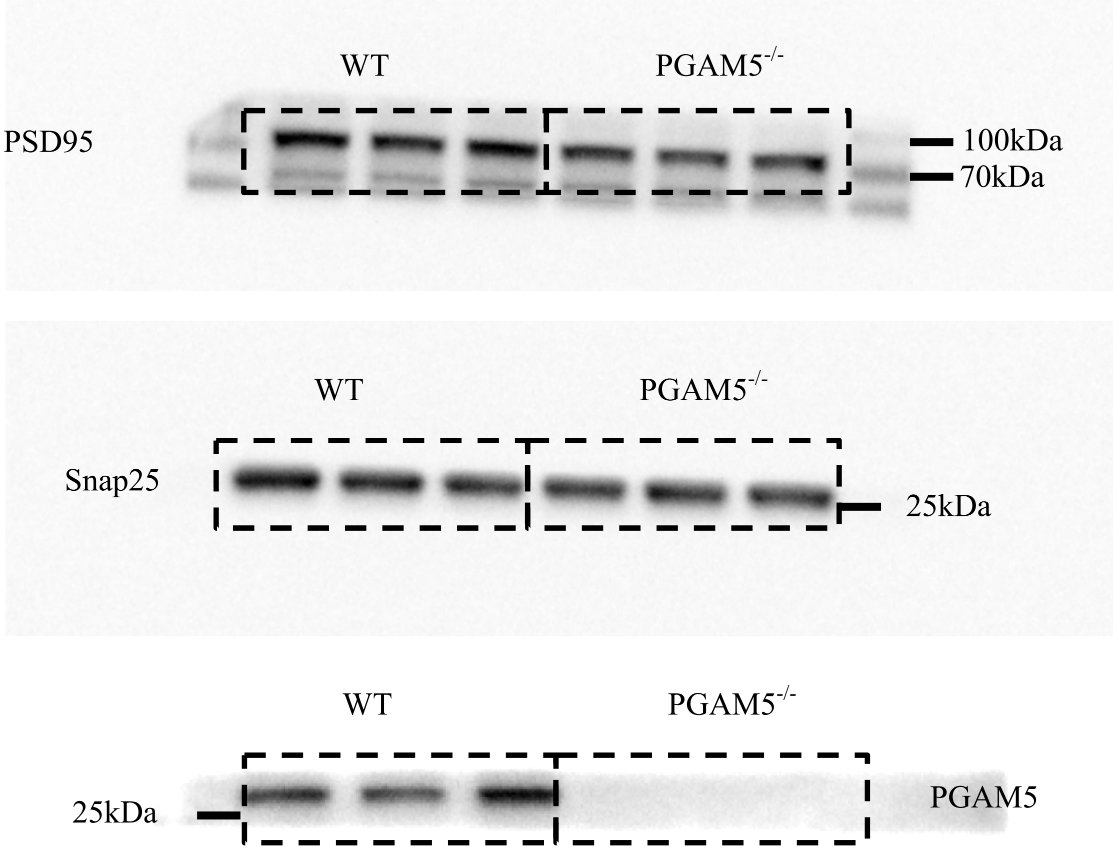

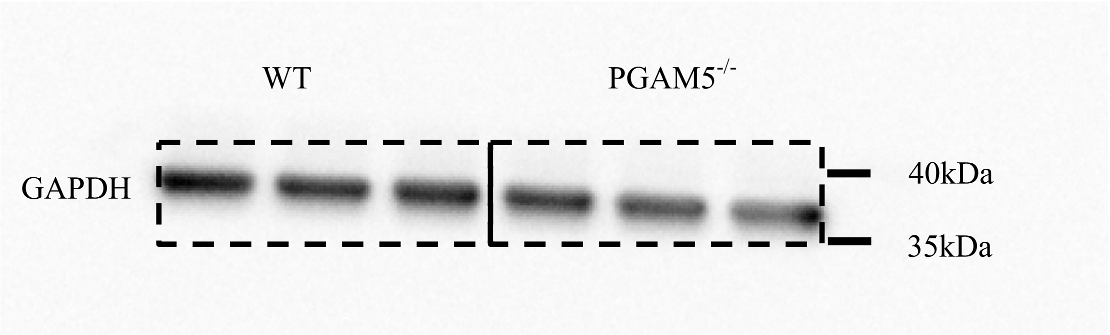

Full unedited blot for Figure4F

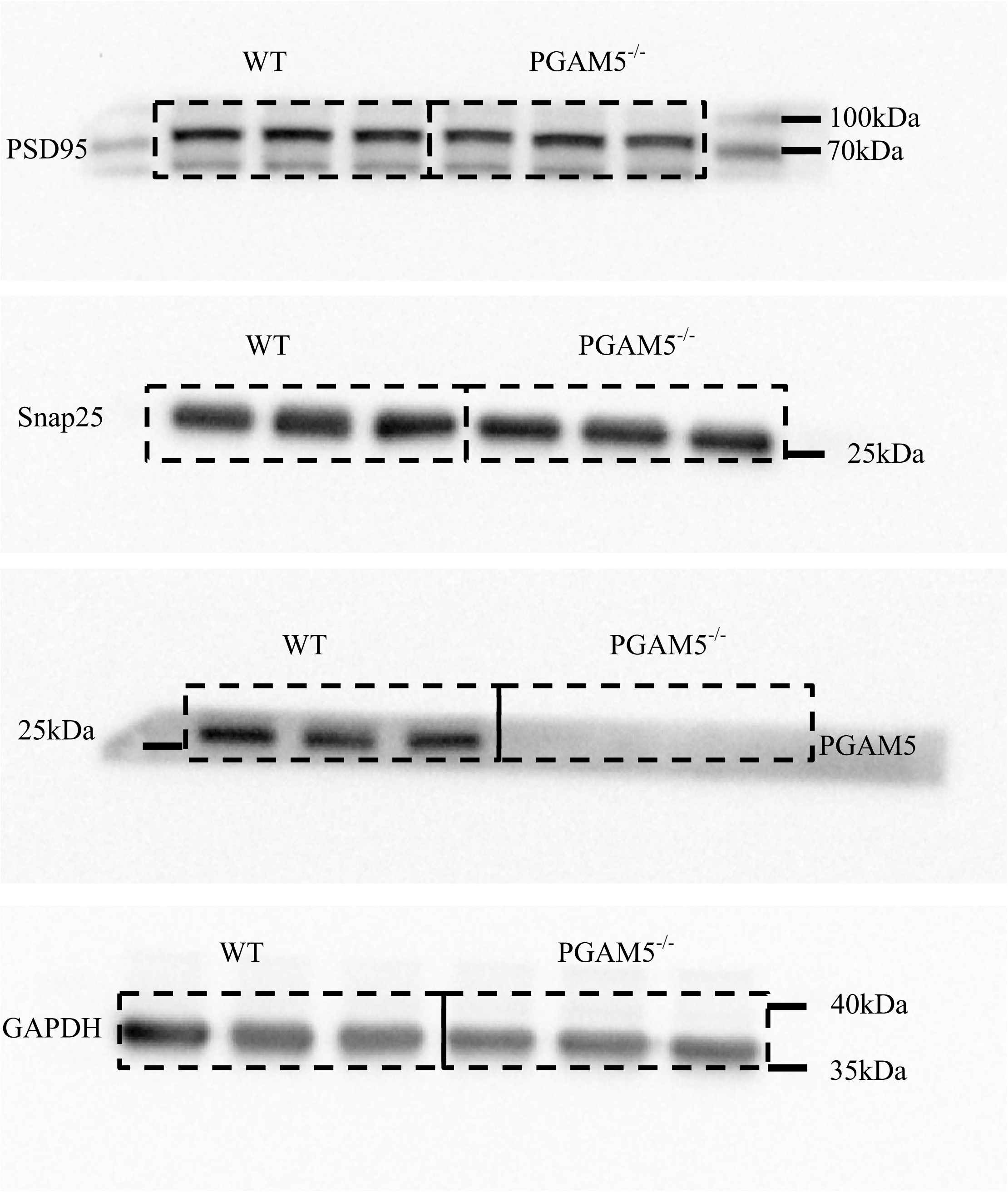

Full unedited blot for Figure5B

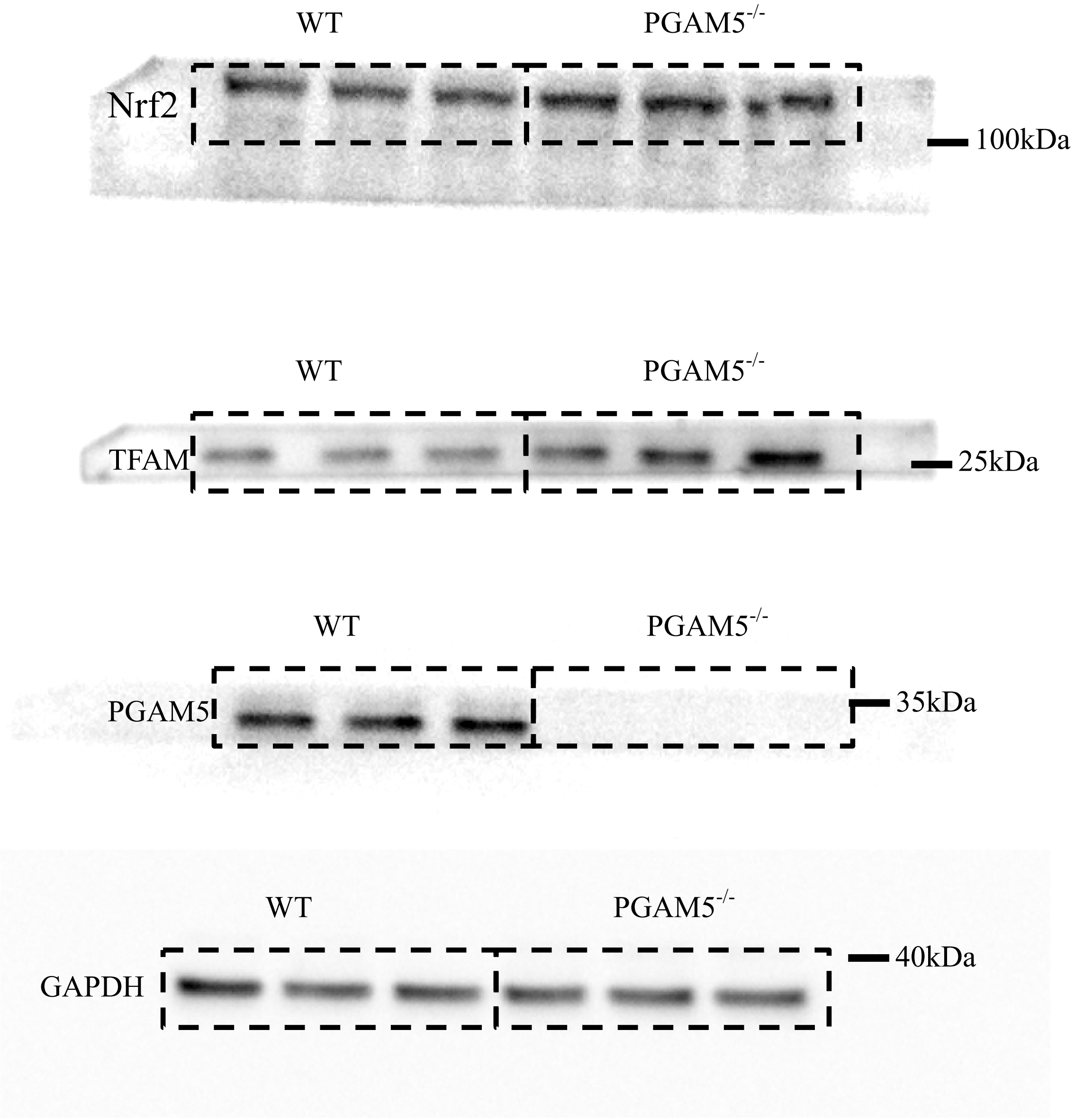

Full unedited blot for Figure5C

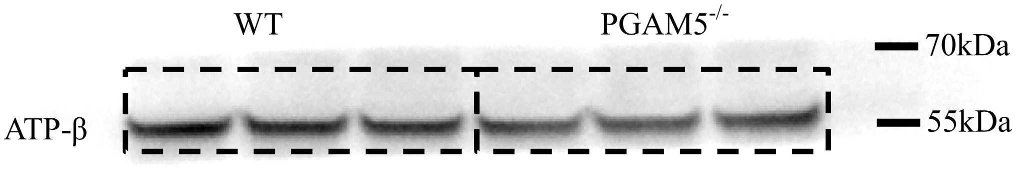

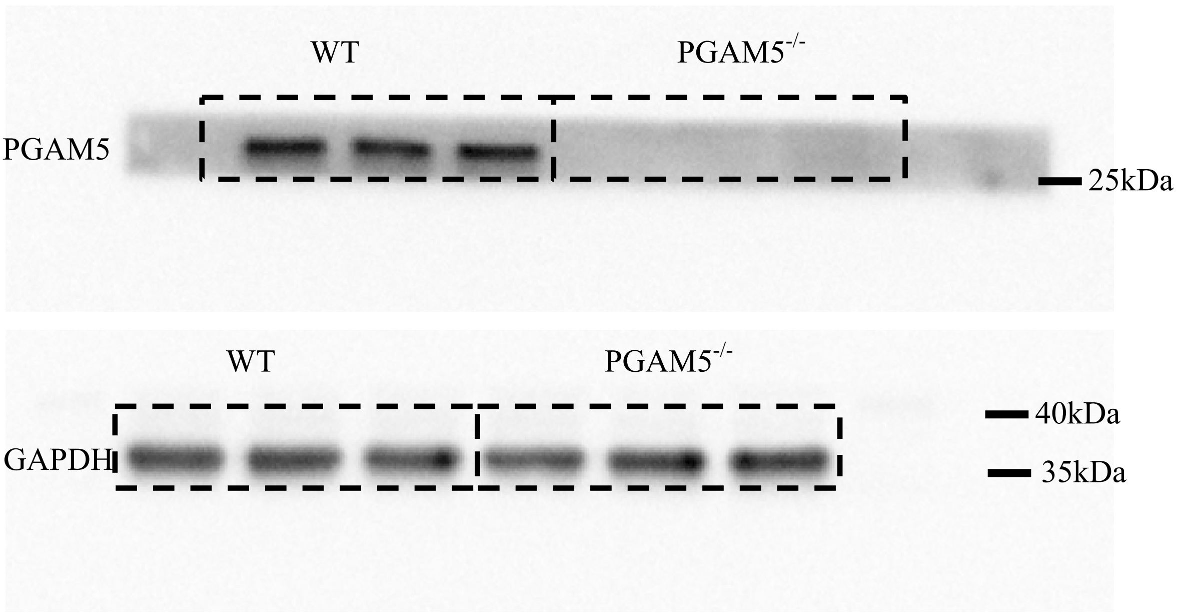

Full unedited blot for Figure5D

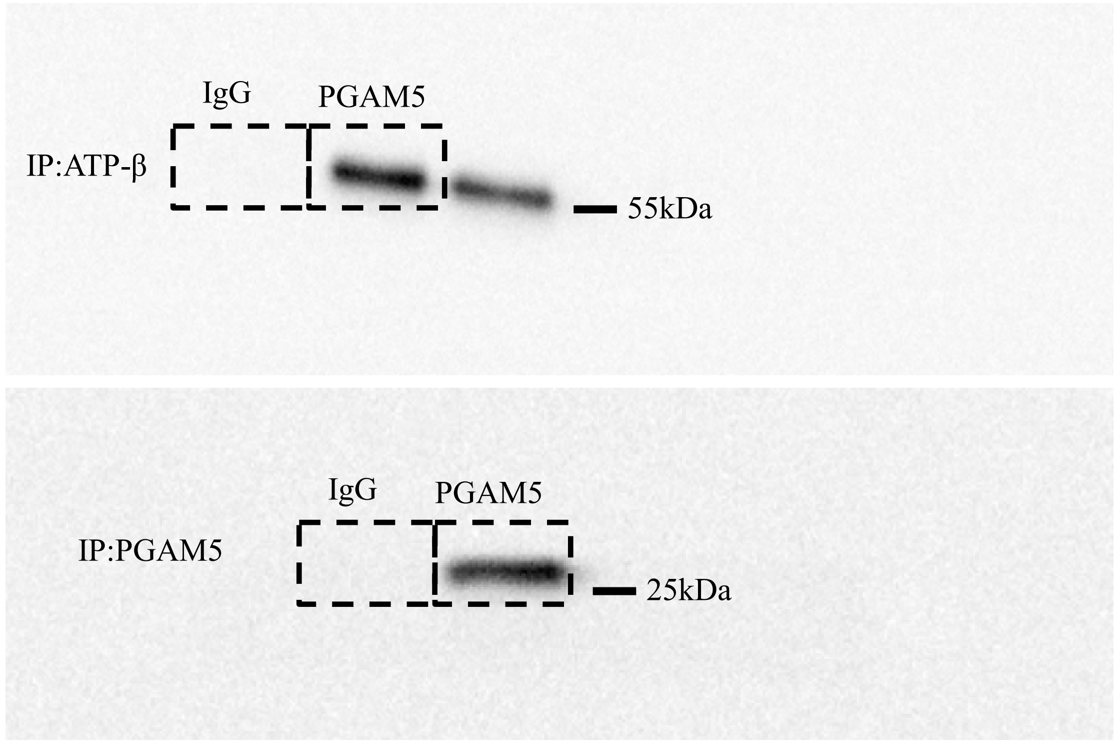

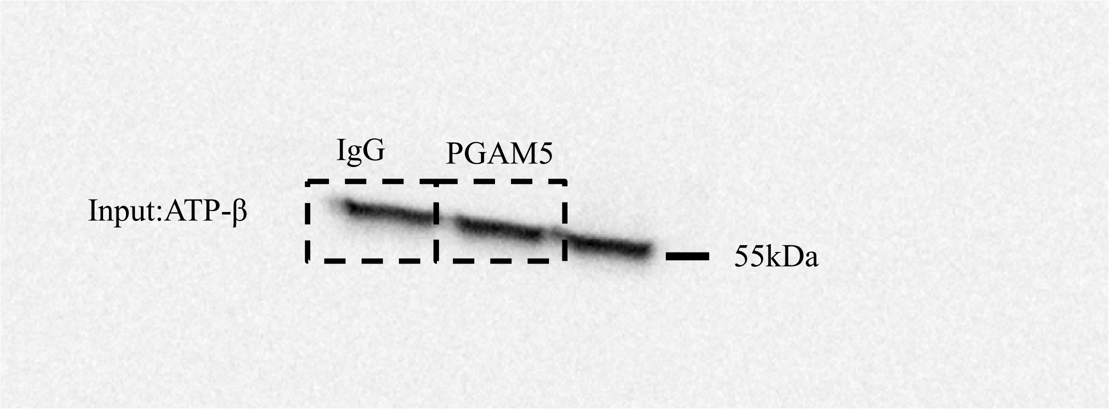

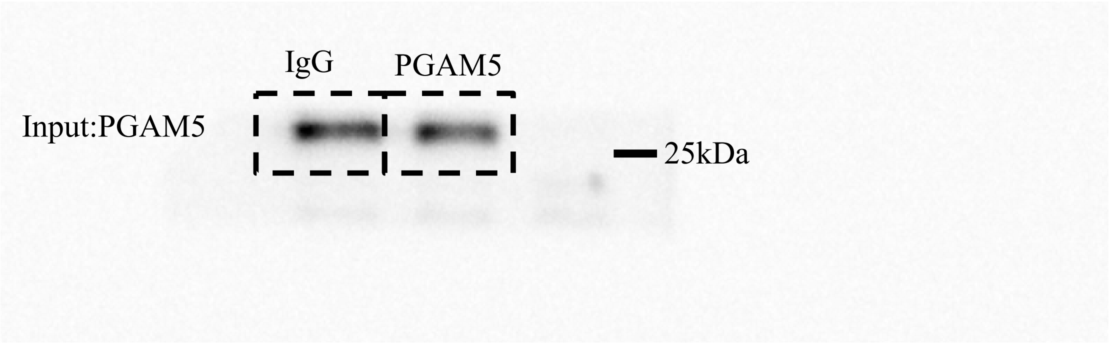

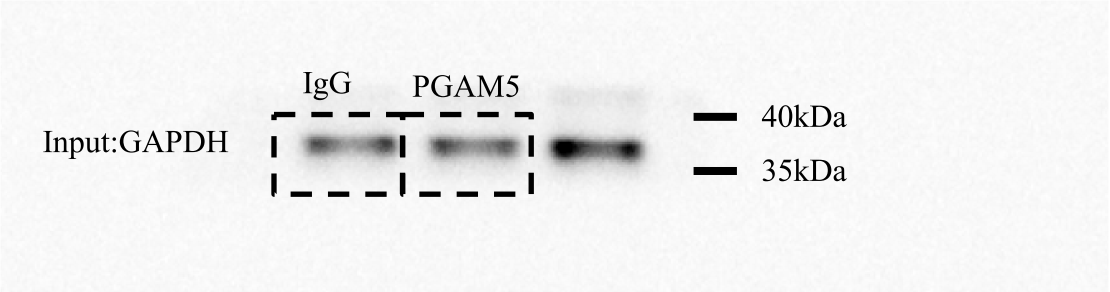

Full unedited blot for Figure5E

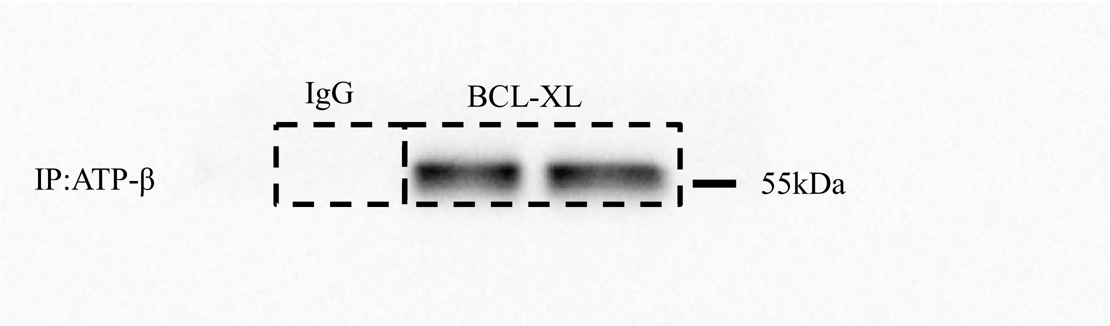

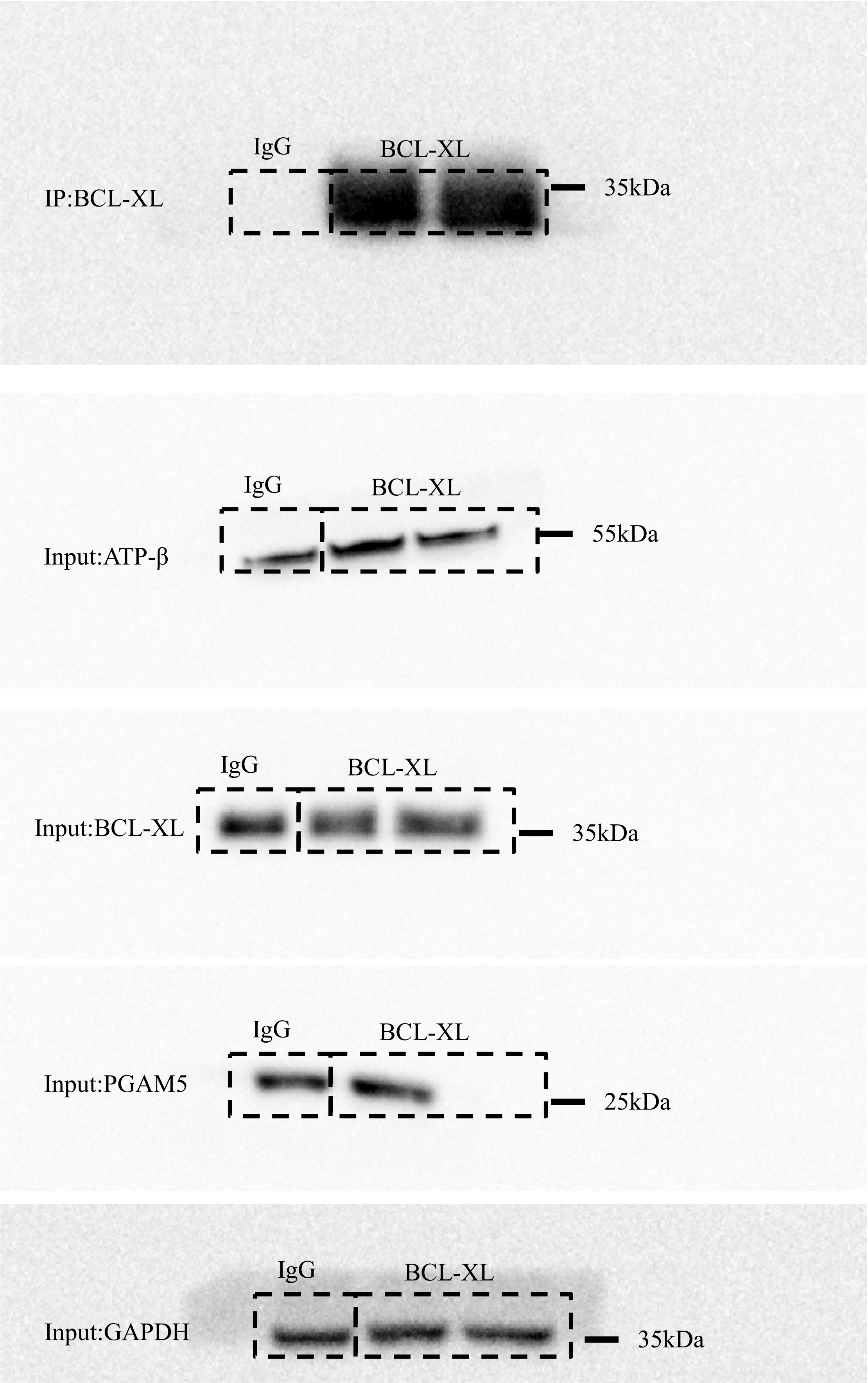
IgG
BCL-XL
IP:BCL-XL
35kDa
IgG
BCL-XL
Input:ATP-β
55kDa
IgG
BCL-XL
Input:BCL-XL
35kDa
IgG
BCL-XL
Input:PGAM5
25kDa
IgG
BCL-XL
Input:GAPDH
35kDa

Full unedited blot for Figure6E

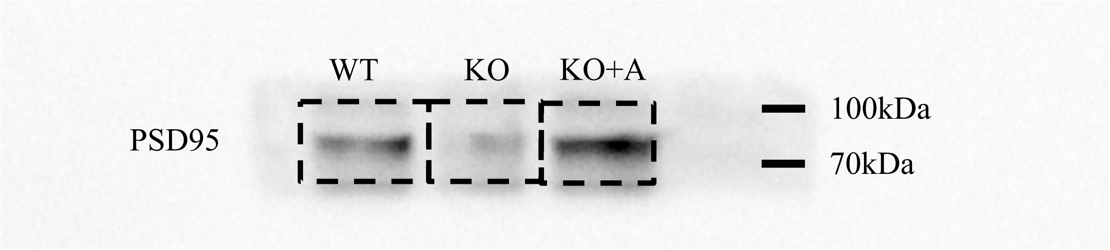

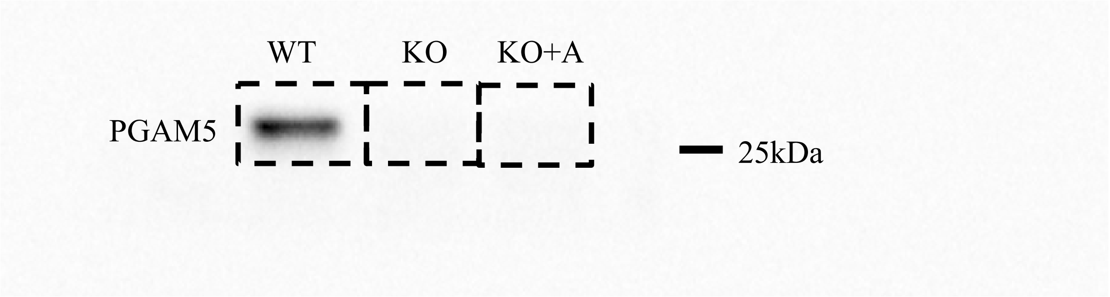

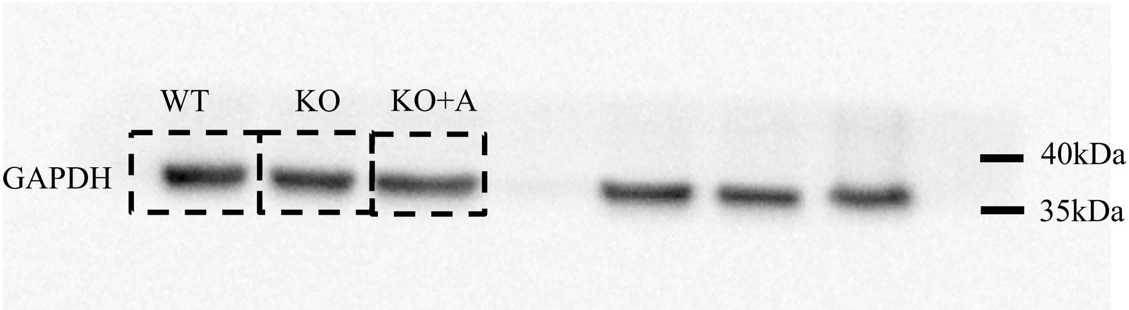

Supplement: Supplementary file 1 — Data S1. [file CNS-30-e14377-s002.pdf]
