# Supplementary material for: PGAM5 knockout causes depressive‐like behaviors in mice via ATP deficiency in the prefrontal cortex
Source: CNS Neurosci Ther. 2023 Aug 25;30(2):e14377. doi: 10.1111/cns.14377 (PMC10848067; doi:10.1111/cns.14377)
Supplement: Supplementary file 2 — Table S1. [file CNS-30-e14377-s001.docx]

**PGAM5 Knockout Causes Depressive-like Behaviors in Mice via ATP-deficiency in the Prefrontal Cortex**

**Supplementary Table S1: summary of statistical results**

|  | Variable | Statistical analysis | *Post hoc* comparisons |
| --- | --- | --- | --- |
| *Figure1* | Total Walking Distance  (Fig. 1A) | Student's *t*-test  Experimental group: T_16_=2.023; p=0.0602 | / |
|  | Center Walking Distance  (Fig. 1B) | Student's *t*-test  Experimental group: T_16=_2.161; p=0.0462 | / |
|  | Immobility Time  (Fig. 1D) | Student's *t*-test  Experimental group: T_16=_3.096; p=0.0069 | / |
|  | relative expression  (normalized to β-actin)  (Fig. 1E-PFC) | Student's *t*-test  Experimental group: T_4=_15.42; p=0.0001 | / |
|  | relative expression  (normalized to β-actin)  (Fig. 1E-HIP) | Student's *t*-test  Experimental group: T_4=_4.756; p=0.0089 | / |
|  | relative expression  (normalized to β-actin)  (Fig. 1E-Striatum) | Student's *t*-test  Experimental group: T_4=_5.953; p=0.004 | / |
|  | Pgam5/Gapdh  (Relative intensity)  (Fig. 1F-PFC) | Student's *t*-test  Experimental group: T_4=_4.239; p=0.0133 | / |
|  | Pgam5/Gapdh  (Relative intensity)  (Fig. 1G-HIP) | Student's *t*-test  Experimental group: T_4=_8.865; p=0.0009 | / |
|  | Pgam5/Gapdh  (Relative intensity)  (Fig. 1H-Striatum) | Mann-whitney test  Experimental group: U_4_<0.0001;  p<0.05 | / |
| *Figure2* | Total Walking Distance  (Fig. 2A) | Student's *t*-test  Experimental group: T_26=_0.9110; p=0.3707 | / |
|  | Center Walking Distance  (Fig. 2B) | Student's *t*-test  Experimental group: T_26=_2.497; p=0.0192 | / |
|  | Immobility Time  (Fig. 2D) | Student's *t*-test  Experimental group: T_26=_4.473; p=0.0001 | / |
|  | Time in open arm  (Fig. 2E) | Student's *t*-test  Experimental group: T_23=_2.198; p=0.0383 | / |
|  | Time in close arm  (Fig. 2F) | Student's *t*-test  Experimental group: T_23=_2.130; p=0.0441 | / |
| *Figure3* | FC Z value  (Fig. 3C-Hip_R) | Student's *t*-test  Experimental group: T_18=_2.584; p=0.0187 | / |
|  | FC Z value  (Fig. 3C-Hip_L) | Student's *t*-test  Experimental group: T_18=_2.637; p=0.0167 | / |
|  | FC Z value  (Fig. 3C-Striatum_R) | Student's *t*-test  Experimental group: T_18=_2.044; p=0.0559 | / |
|  | FC Z value  (Fig. 3C-Striatum_L) | Student's *t*-test  Experimental group: T_18=_2.073; p=0.0528 | / |
|  | FC Z value  (Fig. 3C-GPS_R) | Student's *t*-test  Experimental group: T_18=_2.331; p=0.0316 | / |
|  | FC Z value  (Fig. 3C-GPS_L) | Student's *t*-test  Experimental group: T_18=_2.215; p=0.0399 | / |
|  | FC Z value  (Fig. 3C-Amy_R) | Student's *t*-test  Experimental group: T_18=_2.007; p=0.06 | / |
|  | FC Z value  (Fig. 3C-Amy_L) | Student's *t*-test  Experimental group: T_18=_2.449; p=0.0248 | / |
|  | FC Z value  (Fig. 3C-NAc_R) | Student's *t*-test  Experimental group: T_18=_1.042; p=0.3112 | / |
|  | FC Z value  (Fig. 3C-NAc_L) | Student's *t*-test  Experimental group: T_18=_1.085; p=0.2922 | / |
|  | FC Z value  (Fig. 3C-POAH_R) | Student's *t*-test  Experimental group: T_18=_2.414; p=0.0267 | / |
|  | FC Z value  (Fig. 3C-POAH_L) | Student's *t*-test  Experimental group: T_18=_2.353; p=0.0302 | / |
|  | FC Z value  (Fig. 3C-VTD) | Student's *t*-test  Experimental group: T_18=_1.749; p=0.0973 | / |
| *Figure4* | Spine density  (Fig. 4A) | Student's *t*-test  Experimental group: T_8=_5.815; p=0.0004 | / |
|  | Spine density  (Fig. 4B) | Student's *t*-test  Experimental group: T_8=_1.170; p=0.2756 | / |
|  | Number of Nissl cells  (Fig. 4C) | Student's *t*-test  Experimental group:T_10=_1.265; p=0.2346 | / |
|  | Number of Nissl cells  (Fig. 4D) | Student's *t*-test  Experimental group:T_7=_0.4358; p=0.6761 | / |
|  | Psd95/Gapdh  (Relative intensity)  (Fig. 4E- Psd95 in PFC) | Student's *t*-test  Experimental group:T_4=_6.551; p=0.0028 | / |
|  | Snap25/Gapdh  (Relative intensity)  (Fig. 4E-Snap25 in PFC) | Mann-whitney test  Experimental group: U_4_=2.000;  P=0.275 | / |
|  | Psd95/Gapdh  (Relative intensity)  (Fig. 4F- Psd95 in HIP) | Student's *t*-test  Experimental group:T_4=_0.4395; p=0.6830 | / |
|  | Snap25/Gapdh  (Relative intensity)  (Fig. 4F-Snap25 in HIP) | Student's *t*-test  Experimental group:T_4=_0.8672; p=0.4347 | / |
| *Figure5* | ATP relative value  (Fig. 5A-PFC) | Mann-whitney test  Experimental group: U_4_<0.0001;  p<0.05 | / |
|  | ATP relative value  (Fig. 5A-HIP) | Student's *t*-test  Experimental group:T_4=_2.314; p=0.0816 | / |
|  | Tfam/Gapdh  (Relative intensity)  (Fig. 5B) | Student's *t*-test  Experimental group:T_4=_5.164; p=0.0067 | / |
|  | Nrf2/Gapdh  (Relative intensity)  (Fig. 5B) | Mann-whitney test  Experimental group: U_4_<0.0001;  p<0.05 | / |
|  | ATPβ/Gapdh  (Relative intensity)  (Fig. 5C) | Student's *t*-test  Experimental group:T_4=_1.956; p=0.1221 | / |
| *Figure6* | Total Walking Distance  (Fig. 6A) | One-way ANOVA  Experimental group: f_2,14_=4.816;  p=0.0256 | Dunnett’s test:  KO:  - vs WT: p=0.0143  - vs KO+A:p=0.1703 |
|  | Center Walking Distance  (Fig. 6A) | One-way ANOVA  Experimental group: f_2,14_=8.972;  p=0.0031 | Dunnett’s test:  KO:  - vs WT: p=0.0030  - vs KO+A:p=0.0065 |
|  | Immobility Time  (Fig. 6B) | One-way ANOVA  Experimental group: F_2,15_=16.85; p=0.0001 | Dunnett’s test  KO:  - vs WT: p=0.0003  - vs KO+A:p=0.0003 |
|  | Spine density  (Fig. 6D) | One-way ANOVA  Experimental group: F_2,11_=3.874; p=0.0532 | / |

Abbreviations: PFC:prefrontal cortex; HIP:hippocampus; HIP_R:right HIP; HIP_L:left HIP; GPS_R:right globus pallidus; GPS_L:left globus pallidus; POAH_R:right hypothalamus; POAH_L:left hypothalamus; Amy_R:right amygdala; Amy_L:left amygdala; NAc_R:right nucleus accumbens; NAc_L:left nucleus accumbens; Stria_R:right striatum; Stria_L:left striatum; VTD: ventral tegmental decussation; FC:functional connectivity; WT: wildtype. KO: PGAM5^-/-^. KO+A: PGAM5^-/-^+ATP.
